# Pulmonary atelectasis in newborns with clinically treatable diseases who are on mechanical ventilation: clinical and radiological aspects

**DOI:** 10.1590/0100-3984.2016.0157

**Published:** 2018

**Authors:** Mariana Chiaradia Dominguez, Beatriz Regina Alvares

**Affiliations:** 1 MD, graduate of the Faculdade de Ciências Médicas da Universidade Estadual de Campinas (FCM-Unicamp), Campinas, SP, Brazil.; 2 Assistant Professor in the Radiology Department of the Faculdade de Ciências Médicas da Universidade Estadual de Campinas (FCM-Unicamp), Campinas, SP, Brazil.

**Keywords:** Pulmonary atelectasis/diagnostic imaging, Respiration, artificial, Infant, newborn, Premature birth, Infant, low birth weight

## Abstract

**Objective:**

To analyze the radiological aspects of pulmonary atelectasis in newborns on mechanical ventilation and treated in an intensive care unit, associating the characteristics of atelectasis with the positioning of the head and endotracheal tube seen on the chest X-ray, as well as with the clinical variables.

**Materials and Methods:**

This was a retrospective cross-sectional study of 60 newborns treated between 1985 and 2015. Data were collected from medical records and radiology reports. To identify associations between variables, we used Fisher's exact test. The level of significance was set at *p* < 0.05.

**Results:**

The clinical characteristics associated with improper positioning of the endotracheal tube were prematurity and a birth weight of less than 1000 g. Among the newborns evaluated, the most common comorbidity was hyaline membrane disease. Atelectasis was seen most frequently in the right upper lobe, although cases of total atelectasis were more common in the left lung. Malpositioning of the head showed a trend toward an association with atelectasis in the left upper lobe.

**Conclusion:**

Pulmonary atelectasis is a common complication in newborns on mechanical ventilation. Radiological evaluation of the endotracheal tube placement provides relevant information for the early correction of this condition.

## INTRODUCTION

Pulmonary atelectasis consists of pulmonary collapse, accompanied by hypoventilation; it can affect a lobe, segment, or all of the lung, resulting in a decrease in the ventilation/perfusion ratio^(^^1^^)^. Its incidence varies according to the age and characteristics of the population studied^(^^2^^)^.

The anatomy and physiology of neonates differ in many respects from those of adults ^(^^3^^)^. In neonates, atelectasis is more common because the pulmonary parenchyma is not yet fully formed, undergoing the remodeling that will finalize the development of the capillaries and alveoli^(^^4^^)^.

In preterm neonates, factors that can contribute to the development of atelectasis include the immaturity of the pulmonary parenchyma, the high complacency of the chest cavity, and surfactant deficiency^(^^5^^)^. These are just some of the reasons why lung diseases are quite common in preterm neonates and are the leading cause of death in the neonatal period^(^^6^^)^.

The most common causes of atelectasis in the neonatal period are respiratory distress syndrome, bacterial pneumonia, meconium aspiration syndrome, gastroesophageal reflux, bronchopulmonary dysplasia, pleural effusion, and pneumothorax. Therefore, in order to identify the cause of atelectasis in neonates, it is important to evaluate the clinical data together with the radiological findings^(^^7^^)^.

Mechanical ventilation, used in preterm neonates, as well as in those with neonatal respiratory distress syndrome or hyaline membrane disease, is also a contributing factor in the development of atelectasis, especially when there is improper placement of the endotracheal tube. In such cases, the distal portion of the endotracheal tube can enter the right bronchus, which is more vertical than the left bronchus, resulting in selective intubation and preventing ventilation of the contralateral lung, in which atelectasis can develop because of hypoventilation or the accumulation of secretion. Therefore, attention must be paid to the correct positioning of the endotracheal tube, mainly the depth to which it is inserted^(^^5,8-10^^)^. Movement of the head of the neonate can cause the endotracheal tube to be placed improperly. The endotracheal tube moves caudally when the neck is flexed and cranially when the neck is extended, potentially resulting in extubation or selective intubation of one of the lungs, a mechanism that can occur even during the positioning of the neonate for procedures, such as during X-rays. That is because the movement of the neck resembles that of a crank, for which the endotracheal tube becomes the pivot point^(^^11,12^^)^.

In patients with atelectasis, the physical findings are generally nonspecific and depend on the extent of the affected area. Such patients can present with a variety of symptoms, including cyanosis, coughing, decreased breath sounds on auscultation, and increased work of breathing. Chest X-ray is considered the only safe means of verifying the position of the endotracheal tube; studying the characteristics of the thorax of the neonate facilitates an accurate diagnosis and appropriate clinical follow-up^(^^5,7,8,11^^)^.

In the radiological examination, the areas of atelectasis present increased density due to the reduction of volume. The adjacent areas are hyperlucent, because the uncollapsed regions distend and occupy the lung volume lost to the atelectasis. Atelectasis can also appear in a specific lung lobe, because of the relationships that the lung has with the heart, diaphragm, and pulmonary fissures. Therefore, the collapse of the lower lobes can cause the adjacent contour of the diaphragm to become more opaque. In the middle lobe and lingula, atelectasis can obliterate the right and left heart contours, respectively, and involvement of the right upper lobe can elevate the horizontal fissure. When complete atelectasis of a lung occurs, ipsilateral mediastinal deviation can also be seen^(^^13,14^^)^.

The objective of the present study was to analyze the radiological aspects of atelectasis in neonates with clinically treatable lung diseases. To that end, we evaluated neonates submitted to mechanical ventilation in a neonatal intensive care unit, associating the clinical variables with the characteristics of atelectasis, as well as with the position of the head and of the endotracheal tube on chest X-rays.

## MATERIALS AND METHODS

The project was approved by the Human Research Ethics Committee of the Faculdade de Ciências Médicas da Universidade Estadual de Campinas (Ruling No. 632,769). This was a cross-sectional, retrospective study involving 60 neonates treated in a neonatal intensive care unit between 1985 and 2015. The cases were selected by reviewing the radiological examinations of preterm and full-term neonates with clinically treatable lung diseases who were submitted to mechanical ventilation and diagnosed with atelectasis on the basis of the radiological findings.

Chest X-rays were obtained with a mobile X-ray system (VMX plus; General Electric, Milwaukee, WI, USA), in an anteroposterior view, with vertical beams and with the neonate in the supine position.

All examinations were analyzed by a radiologist with experience in neonatal radiology. The following radiological variables were analyzed: the location and extent of atelectasis; the placement of the endotracheal tube; and the positioning of the head of the neonate.

Clinical data were collected through a review of medical records and radiology reports, as well as through communication with the attending radiologist. The following clinical data were collected: gestational age at birth; birth weight; the reason for mechanical ventilation; the 5-min Apgar score; chronological age; and type of delivery.

For quality control (to check for inconsistencies in data entry), the data obtained were double entered into a specific Microsoft Excel database, after which they were organized and stored. The categorical variables were analyzed by descriptive statistics, expressed as absolute and relative frequencies. To identify associations between variables, we used Fisher's exact test. The level of significance adopted was *p* < 0.05.

## RESULTS

Of the 60 neonates evaluated, 31 (51.7%) were male, 28 (46.7%) were female, and 1 (1.7%) was of indeterminate sex. Preterm neonates predominated, 51 (85.0%) of the 60 neonates having been born at a gestational age of less than 37 weeks. The birth weight was less than 1000 g in 34 (56.7%) neonates, 1000-1500 g in 15 (25.0%), and greater than 1500 g in 11 (18.3%).

Among the neonates evaluated, the most common clinical disease was hyaline membrane disease, observed in 41 (68.3%) neonates. In addition, 25 (41.7%) neonates developed sepsis and 11 (18.3%) had bronchial dysplasia. The clinical variables evaluated are shown in Table 1.

**Table 1:** Clinical variables.

Variable	N	%
Sex		
Male	31	51.7
Female	28	46.7
Indeterminate	1	1.7
Gestational age		
< 37 weeks	51	85.0
≥ 37 weeks	9	15.0
Birth weight		
< 1000 g	34	56.7
1000-1500 g	15	25.0
> 1500 g	11	18.3
Type of delivery		
Vaginal	22	36.7
Cesarean	38	63.3
Clinical condition		
Hyaline membrane disease	41	68.3
Sepsis	25	41.7
Pneumonia	14	23.3
Bronchial dysplasia	11	18.3
Transitory tachypnea	10	16.7
Meconium aspiration syndrome	4	6.7

**Figure 1:**
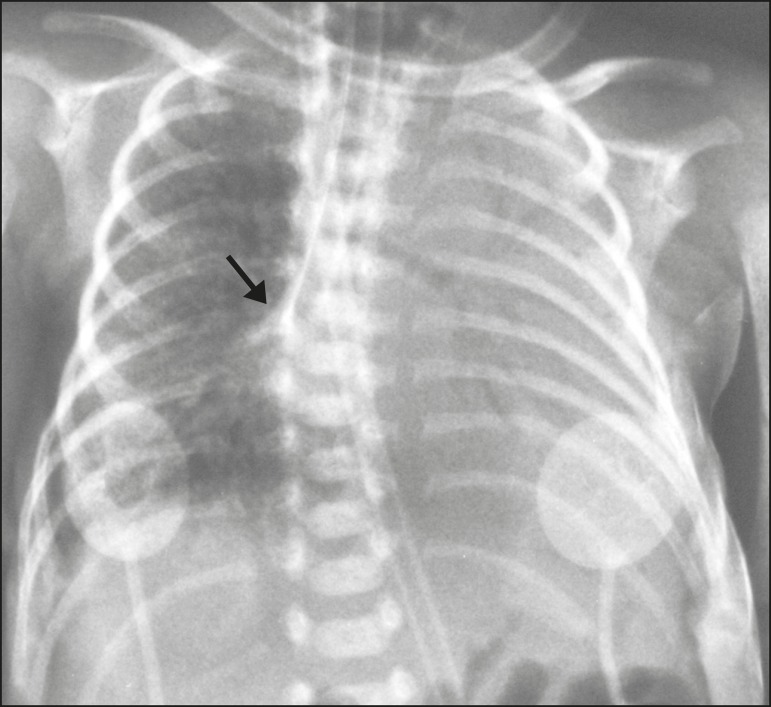
Chest X-ray of a neonate born at 29 weeks of gestation with a birth weight of 745 g, showing complete atelectasis of the left lung resulting from selective intubation, the distal end of the endotracheal tube being located in the right bronchus (arrow).

**Figure 2:**
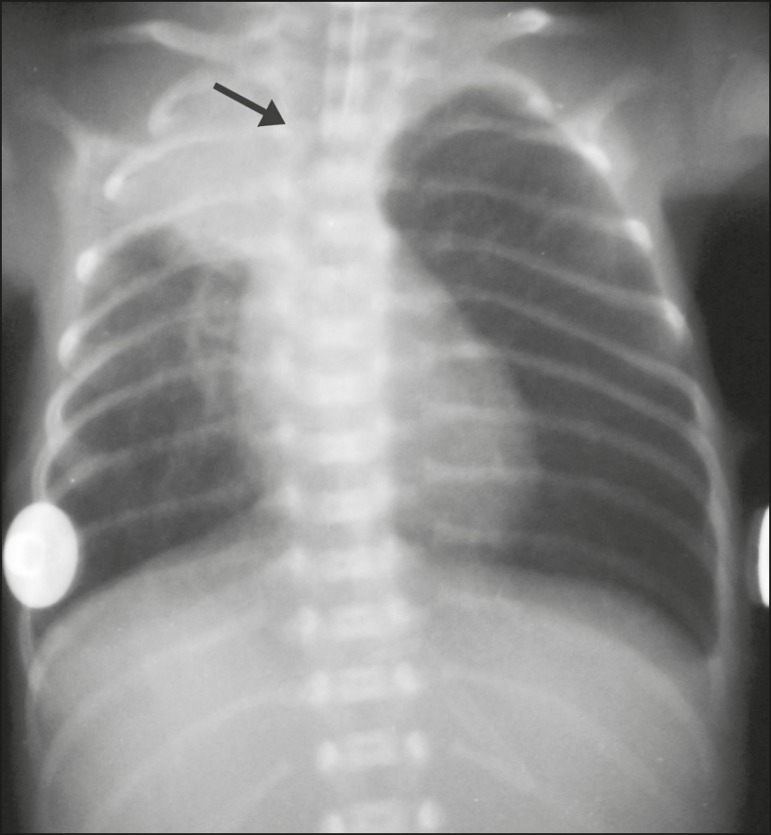
Chest X-ray of a neonate born at 25 weeks of gestation with a birth weight of 900 g, showing atelectasis of the right upper lobe. The endotracheal tube is positioned correctly (arrow).

**Figure 3:**
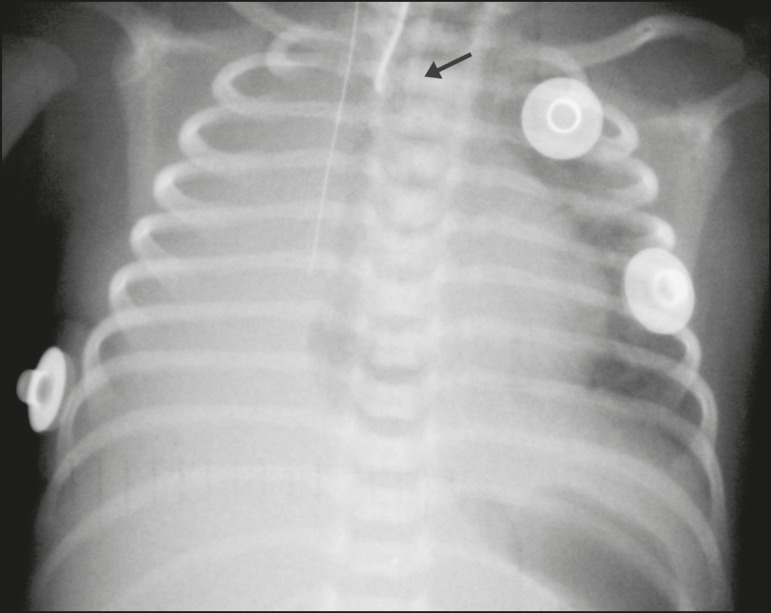
Chest X-ray of a neonate born at 33 weeks of gestation with a birth weight of 1400 g, showing complete atelectasis of the right lung, with a wellpositioned endotracheal tube (arrow).

The analysis of the positioning of the endotracheal tube revealed improper placement in 52 (86.7%) of the 60 X-rays evaluated. The endotracheal tube was found to have been positioned correctly-at the level of first thoracic vertebra (T1)-in 8 (13.3%) cases. As illustrated in Figures 1, 2, and 3, selective intubation was observed in 6 (10%) cases. The endotracheal tube was found to have been positioned below the ideal (T1) level in 41 cases (68.3%), compared with 11 (18.3%) in which it was positioned above the ideal level (Table 2).

**Table 2:** Position of the endotracheal tube, as seen on chest X-rays of neonates.

Position of the endotracheal tube	N	%
C6	4	6.7
C7	4	6.7
C6-C7	3	5.0
T1*	8	13.3
T2	5	8.3
T3	6	10.0
T4	1	1.7
T5	4	6.7
T6	1	1.7
T1-T2	3	5.0
T2-T3	4	6.7
T3-T4	3	5.0
T4-T5	5	8.3
T5-T6	2	3.3
T6-T7	1	1.7
Right main bronchus	2	3.3
Right intermediate bronchus	4	6.7

* Appropriate position.

Improper endotracheal tube placement was associated (by Fisher's exact test) with the following clinical variables (Table 3): gestational age of less than 37 weeks; and birth weight of less than 1000 g.

**Table 3:** Association between the positioning of the endotracheal tube and the clinical variables evaluated.

	Endotracheal tube position		
Clinical variable	Correct	Incorrect	P
Gestational age			
< 37 weeks	3	48	0.001
≥ 37 weeks	5	4	
Weight			
< 1000 g	3	31	0.0033
1000-1500 g	0	15	
> 1500 g	5	6	
Sex			
Male	5	26	0.4281
Female	2	26	
Type of delivery			
Cesarean	5	33	1.000
Vaginal	3	19	

Of the 60 neonates evaluated, 19 (31.7%) presented a collapsed lobe in the left lung. Among those 19 cases, complete atelectasis of the left lung occurred in 16 (84.2%). In the right lung, a collapsed lobe was seen in 54 (90.0%) neonates, 12 (22.2%) of whom had complete right-lung atelectasis.

In 50 cases (83.3%), the X-ray showed that the head of the neonate was well positioned. There was a trend toward an association between poor positioning of the head and the occurrence of atelectasis in the upper left lobe (*p* = 0.052), although there was no such association with the occurrence of atelectasis in other lobes (Table 4).

**Table 4:** Association between the positioning of the head of the neonate and the site of atelectasis.

	Head of the neonate		
Lung site	Correct	Incorrect	P
Right upper lobe			
No	6	2	0.609
Yes	44	8	
Right middle lobe			
No	38	8	1.000
Yes	12	2	
Right lower lobe			
No	38	7	0.699
Yes	12	3	
Left upper lobe			
No	38	4	0.052
Yes	12	6	
Left lower lobe			
No	38	5	0.128
Yes	12	5	

## DISCUSSION

In the radiology literature of Brazil, interest in studies in the field of pediatrics has been growing^(^^15-24^^)^. Atelectasis is a common condition in neonates on mechanical ventilation, being the main complication of that type of ventilatory support^(^^25-27^^)^.

Data in the literature indicate that 42% of neonates on mechanical ventilation develop atelectasis^(^^28^^)^, which can occur when the endotracheal tube is either above or below the correct position. In adults admitted to the intensive care unit, atelectasis reportedly occurs most often in the lower lobes. Because of the anatomical differences between pediatric and adult patients, together with the greater difficulty in positioning the endotracheal tube in the former, atelectasis in pediatric patients typically occurs in the superior lobes, especially the right upper lobe. Complete atelectasis is more common when there is total obstruction of the main bronchus, either by a mucus plug or a poorly positioned endotracheal tube. Because selective intubation usually occurs in the right bronchus, hypoventilation and the consequent atelectasis usually appears in the left lung^(^^28,29^^)^, which explains the higher frequency of complete atelectasis of the left lung observed in our study.

The distal end of the endotracheal tube should be positioned at the T1 level, because it is an anatomical landmark that can be used for neonates of any gestational age and is easily visualized on X-rays^(^^30^^)^. Once the incorrect placement of the endotracheal tube has been identified by radiological examination, it should be repositioned ^(^^14^^)^. Nevertheless, in approximately 25% of cases, the endotracheal tube remains poorly placed even after being repositioned^(^^31^^)^.

In the present study, involving neonates with atelectasis, we found that the distal end of the endotracheal tube had been positioned correctly (at the T1 level) in 13.3% of the sample. Given that incorrect placement of the endotracheal tube cannot be considered the predisposing factor for atelectasis in these patients, the most probable cause, according to data in the literature, was the increased airway mucosal viscosity, leading to the formation of mucus plugs, which, displaced by bronchial obstruction, caused this complication^(^^32^^)^.

Selective intubation of the right lung, which is considered one of the most serious complications of mechanical ventilation because it is associated with an increased risk of alveolar hyperventilation, pneumothorax, and atelectasis^(^^8,13,14^^)^, occurred in 10% of the cases evaluated in the present study. In a study conducted in a pediatric intensive care unit, Souza et al.^(^^33^^)^ observed selective intubation at an incidence of 20%, intubation of the right bronchus being more common, as was the occurrence of selective intubation in children under 2 years of age. Kuhns et al.^(^^9^^)^, in a study involving infants under 1 year of age, found that the endotracheal tube was positioned below the carina in 18 of the 36 patients evaluated. In 17 of those patients, the endotracheal tube had been placed in the right main bronchus or right intermediate bronchus, having been placed in the left main bronchus in only one patient. In our study, despite the high rate of incorrect endotracheal tube placement, selective intubation was less common than in other studies.

In the cases of incorrect endotracheal tube placement identified in the present study, the endotracheal tubes were repositioned after their incorrect placement was observed on X-rays. However, we did not evaluate follow-up X-rays, because that was not one of the objectives of the study. We found that the rate of incorrect endotracheal tube placement was higher in neonates with a birth weight of less than 1000 g, as well as in those with a gestational age of less than 37 weeks, thus confirming that preterm neonates and neonates with an extremely low birth weight are more susceptible to intubation failure.

The intubation of neonates is a challenge, even with the use of specific methods and protocols. In a study that sought to evaluate the accuracy of the 7-8-9 rule for intubation, incorrect placement of the endotracheal tube, its distal end being located below the ideal point, was found to have occurred in neonates with a birth weight of less than 750 g^(^^34^^)^. Other authors have reported that the use of the 7-8-9 rule without adequate auscultation led to inappropriate endotracheal tube positioning, the distal end being located above the ideal point, in approximately half of all neonates with a birth weight of less than 1000 g^(^^35^^)^, whereas others have reported no significant difference in the rate of incorrect endotracheal tube placement with or without the use of the rule in such infants^(^^36^^)^.

In the present study, despite the low rate of incorrect positioning of the head of the neonate during X-ray examination, that factor showed a trend toward an association with the occurrence of atelectasis in the left upper lobe, an association that has not been established in other studies. That trend might be due to the fact that head movement, during the radiological examination or otherwise, causes endotracheal tube displacement ranging from 0.7 cm to 1.2 cm, predisposing to the occurrence of atelectasis^(^^37-39^^)^. However, because there are no data in the literature regarding such occurrence, further research is needed in order to evaluate this association in greater detail.

On the basis of our findings in the present study, we conclude that atelectasis is a serious complication in neonates submitted to mechanical ventilation, especially those who were born premature and had a very low birth weight. In such neonates, the risk of complications and mortality is increased, the radiological evaluation of the endotracheal tube placement being relevant for the treatment and early correction of atelectasis.
